# Population-Based Surveillance for Invasive Pneumococcal Disease in Homeless Adults in Toronto

**DOI:** 10.1371/journal.pone.0007255

**Published:** 2009-09-29

**Authors:** Agron Plevneshi, Tomislav Svoboda, Irene Armstrong, Gregory J. Tyrrell, Anna Miranda, Karen Green, Donald Low, Allison McGeer, for the Toronto Invasive Bacterial Diseases Network

**Affiliations:** 1 Toronto Invasive Bacterial Diseases Network, Toronto, Ontario, Canada; 2 Department of Microbiology, Mount Sinai Hospital, Toronto, Ontario, Canada; 3 Centre for Research on Inner City Health, St. Michael's Hospital, Toronto, Ontario, Canada; 4 City of Toronto Public Health Department, Toronto, Ontario, Canada; 5 Canadian National Centre for Streptococcus, Edmonton, Alberta, Canada; University of Cape Town, South Africa

## Abstract

**Background:**

Identification of high-risk populations for serious infection due to *S. pneumoniae* will permit appropriately targeted prevention programs.

**Methods:**

We conducted prospective, population-based surveillance for invasive pneumococcal disease and laboratory confirmed pneumococcal pneumonia in homeless adults in Toronto, a Canadian city with a total population of 2.5 M, from January 1, 2002 to December 31, 2006.

**Results:**

We identified 69 cases of invasive pneumococcal disease and 27 cases of laboratory confirmed pneumococcal pneumonia in an estimated population of 5050 homeless adults. The incidence of invasive pneumococcal disease in homeless adults was 273 infections per 100,000 persons per year, compared to 9 per 100,000 persons per year in the general adult population. Homeless persons with invasive pneumococcal disease were younger than other adults (median age 46 years vs 67 years, P<.001), and more likely than other adults to be smokers (95% vs. 31%, P<.001), to abuse alcohol (62% vs 15%, P<.001), and to use intravenous drugs (42% vs 4%, P<.001). Relative to age matched controls, they were more likely to have underlying lung disease (12/69, 17% vs 17/272, 6%, P = .006), but not more likely to be HIV infected (17/69, 25% vs 58/282, 21%, P = .73). The proportion of patients with recurrent disease was five fold higher for homeless than other adults (7/58, 12% vs. 24/943, 2.5%, P<.001). In homeless adults, 28 (32%) of pneumococcal isolates were of serotypes included in the 7-valent conjugate vaccine, 42 (48%) of serotypes included in the 13-valent conjugate vaccine, and 72 (83%) of serotypes included in the 23-valent polysaccharide vaccine. Although no outbreaks of disease were identified in shelters, there was evidence of clustering of serotypes suggestive of transmission of pathogenic strains within the homeless population.

**Conclusions:**

Homeless persons are at high risk of serious pneumococcal infection. Vaccination, physical structure changes or other program to reduce transmission in shelters, harm reduction programs to reduce rates of smoking, alcohol abuse and infection with bloodborne pathogens, and improved treatment programs for HIV infection may all be effective in reducing the risk.

## Introduction

*Streptococcus pneumoniae* is the most common cause of bacterial pneumonia, bacteremia and meningitis in adults, and is a major cause of morbidity and mortality in the general population [1], [2]. Homeless adults may be at greater risk than other adults both because of underlying medical conditions that increase their risk of infection such as chronic liver disease or HIV infection [2]–[8], and because communal living in shelters may be associated with transmission of pathogenic strains [8]–[11]. Authors of at least two reports of clusters of pneumococcal disease in shelters have recommended systematic vaccination of shelter residents [9], [10]. However, no national guidelines currently include such a recommendation, and there are few data addressing the burden of illness associated with pneumococcal infection in the homeless.

The objective of this study was to describe the epidemiology of serious pneumococcal disease in homeless adults in metropolitan Toronto over a five year period.

## Methods

### Population-based surveillance

The Toronto Invasive Bacterial Diseases Network (TIBDN) has conducted prospective, population-based surveillance of invasive pneumococcal disease in metropolitan Toronto, Canada (population, 2.5 million), since 1 January 1995 [12]–[14]. The surveillance network includes all hospital-based laboratories that provide clinical care to area residents. This comprises 25 licensed microbiology laboratories that serve 27 hospitals, long-term care facilities and out-patient offices. Personnel from these laboratories telephone the central TIBDN study office at the Mount Sinai Hospital in Toronto whenever *S. pneumoniae* is isolated from a sterile site or respiratory specimen. No laboratories serving this population used urinary antigen detection for the diagnosis of pneumococcal disease during the surveillance period. For each case, initial demographic data and the pneumococcal isolate are forwarded to the central TIBDN office. Additional clinical data, including patient co-morbidities, clinical course and outcome, antimicrobial therapy in the 3 months before presentation, and outpatient therapy for the current episode before the blood sample was obtained for culture, are acquired by chart review, patient interview, and by contacting the patient's attending physicians. Annual audits are conducted in each laboratory to ensure complete reporting. Surveillance and associated studies are approved by the research ethics boards of all participating institutions. In addition, invasive pneumococcal disease has been reportable in Ontario since January 1, 2001, with all cases of disease reported in Toronto investigated by Toronto Public Health. This study included all adult (> = 15 years of age) cases of invasive pneumococcal disease and laboratory confirmed pneumococcal pneumonia presenting between January 1, 2002 to December 31, 2006, with data from TIBDN and public health pooled to ensure accurate identification of homeless persons.

Population statistics were obtained from Statistics Canada, and annual incidence rates calculated using the estimated population on July 1 of each year. The population of homeless adults was estimated to be 5052, based on a single census conducted in April, 2006 [15].

### Population vaccination uptake

A publicly funded 23-valent polysaccharide vaccination program was introduced into Ontario in 1996 [16]. The seven-valent pneumococcal conjugate vaccine was licensed in Canada in June 2001, with a publicly funded program for infants initiated in Ontario in January 2005 [17].

### Definitions

Persons with invasive pneumococcal disease were classified as homeless if they had no fixed address, or gave their address as an emergency or transitional shelter. [18].

Invasive pneumococcal disease was defined as isolation of *Streptococcus pneumoniae* from a sterile body fluid with a compatible clinical syndrome. Sterile sites included blood, CSF, peritoneal fluid, pleural fluid, or abscess aspirate, but not bronchoalveolar lavage. Non-bacteremic pneumococcal pneumonia was defined as per Musher *et al.* [19], and required: (i) a clinical presentation including symptoms (eg. cough, sputum, fever) and physical findings consistent with pneumonia; (ii) radiographic confirmation of a pulmonary infiltrate; (iii) microscopic examination of a Gram stained sputum with at least moderate numbers of white blood cells per high power field and a predominance of Gram positive cocci in pairs or chains; (iv) a sputum culture that yielded *S. pneumoniae* but no other respiratory pathogen and (v) blood cultures were obtained and did not yield a pathogen.

### Laboratory methods

All isolates were serotyped at the central study laboratory at the Mount Sinai Hospital, or the National Center for Streptococcus, Edmonton, Canada using commercial antisera (Statens Seruminstitut, Copenhagen, Denmark). Broth microdilution antimicrobial susceptibility testing was performed and interpreted by Clinical and Laboratory Standards Institute standards [20].

### Statistical Methods

All data was entered in duplicate and analyzed using SAS for PC version 9.1 (SAS Institute, Cary, NC). Proportions were compared using chi-square or Fisher's exact tests and odds ratios calculated with 95% confidence intervals. Two comparisons of risk factors for invasive pneumococcal disease were conducted: one in which risk factors in cases occurring homeless adults were compared to all cases in other adults, and one in which cases in homeless adults were compared a cohort of other adults constructed by identifying, for each case in a homeless person, the four cases of disease in housed persons closest in age.

## Results

### Incidence

Over the five year period, there were 69 episodes of invasive pneumococcal disease in homeless persons, and 970 episodes in other residents of Toronto. During the time of the study, an estimated 5050 adults were homeless on any given day, with an estimated 27,000 persons homeless over the course of a year. Thus, the estimated rate of invasive disease in homeless persons was 273 per 100,000 per year, 30 fold higher than the concurrent rate in housed adults (9.0 per 100,000 per year). Homeless persons comprised 6.6% of all cases of invasive pneumococcal disease, but only 0.2% of the population of Toronto.

**Figure 1:**
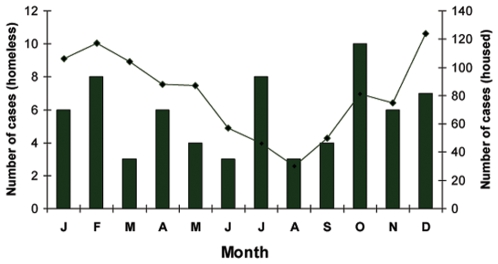
Number of cases of invasive pneumococcal disease, by month, in homless and housed residents of Toronto, 2002–2006. Bars represent cases in homeless persons, line represents cases in housed persons. The proportion of infections in homeless persons was significantly greater in summer (14/137, 10.2%) than in fall (23/305, 7.5%), winter (17/345, 4.9%) or spring (13/246, 5.3%), P = .05.

The typical seasonal pattern of invasive pneumococcal disease in Toronto, with highest rates in the winter months, and a nadir in July and August, was not present in cases in homeless persons (Figure 1). The higher proportion of invasive disease in homeless persons in the summer and fall could not be explained by differences in the age, differences in the occurrence of underlying illness or differences in the serotypes of infecting isolates (data not shown). However, smokers who were not homeless were also more likely to present with invasive pneumococcal disease in the summer months: 48 of 297 (16%) cases of invasive pneumococcal disease in smokers occurred in July and August, compared to 64 of 582 (11%,) in non-smokers, P = .03.

### Clinical characteristics of invasive pneumococcal disease

Characteristics of invasive pneumococcal disease in homeless and housed adults are shown in Table 1. The most significant difference is in age distribution: homeless adults accounted for 3% (1/36) of cases of invasive pneumococcal disease in persons aged <30 years of age, 19% (49/256) of cases in those aged 30–50 years, 6% (10/157) of cases in those aged 50–60 years, and 1.5% (9/590) cases in those aged 60 years and older (P<.001). Homeless persons were more likely than others to be smokers, to abuse alcohol and use intravenous drugs, and to have chronic liver disease (see Table 1). Relative to age matched controls, they were more likely to have underlying lung disease (12/69, 17% vs 17/272, 6%, P = .006), but not more likely to be HIV infected (17/69, 25% vs 58/282, 21%, P = .73).

**Table 1:** Clinical characteristics of episodes of invasive pneumococcal disease in housed and homeless adults, Toronto, 2002–2006.

Characteristic	Homeless persons (N = 69) ‡	All other adults (N = 970)**	P value	Age matched, housed adults (N = 276)** ††	P value
Median age (range)	44.7 years (27–74 y)	66.5 years (15–108 y)	<.001	44.3 years (27–74 y)	.84
Gender (N, % male)	56/69 (81%)	553/970 (57%)	<.001	183/276 (66%)	.02
Underlying illness					
Any*	61/69 (88%)	736/964 (76%)	.09	188/275 (68%)	.001
Diabetes mellitus	5/69 (7.3%)	182/950 (19%)	.02	25/272 (9.2%)	.81
Chronic cardiac disease	12/69 (17%)	302/950 (32%)	.02	33/272 (12%)	.23
Chronic lung disease	12/69 (17%)	193/950 (20%)	.67	17/272 (6.2%)	.006
Cancer	4/69 (5.8%)	195/950 (21%)	.005	32/272 (12%)	.19
Chronic liver disease	26/69 (38%)	75/950 (7.9%)	<.001	35/272 (13%)	<.001
Chronic kidney disease	2/69 (2.9%)	69/950 (7.3%)	.22	11/272 (4.0%)	1.0
HIV infection	17/69 (25%)	80/950 (8.4%)	<.001	58/282 (21%)	.63
Smoker	54/56 (96%)	297/963 (31%)	<.001	125/253 (49%)	<.001
Alcohol abuse	43/69 (62%)	142/950 (15%)	<.001	63/272 (23%)	<.001
Intravenous drug use	29/69 (42%)	35/950 (3.7%)	<.001	25/272 (9.2%)	<.001
Recent antibiotic exposure					
Any antibiotic prior 3 mos†	21/33 (64%)	205/762 (27%)	<.001	58/221 (26%)	<.001
Failing oral therapy§	5/63 (7.9%)	60/848 (7.1%)	.80	16/245 (6.5%)	.78
Clinical diagnosis					
Bacteremic pneumonia	60/69 (87%)	692/949 (73%)	0.08	190/276 (69%)	0.02
Sepsis without focus	5/69 (7.2%)	140/949 (15%)		38/276 (14%)	
Meningitis	2/69 (2.9%)	47/949 (5.0%)		18/276 (6.5%)	
Other	2/69 (2.9%)	70/949 (7.4%)		30/276 (11%)	
Required hospitalization	60/69 (87%)	838/952 (88%)	.94	222/276 (80%)	.54
Hospital-acquired disease	1/69 (1.4%)	49/954 (5.1%)	.25	9/276 (3.5%)	.69
Median length of stay (range)	6 days (1–74 days)	8 days (1–214 days)	.23	6 days (1–214 days)	.96
Outcome/complications					
Empyema	1/69 (1.5%)	36/949 (3.8%)	.51	7/276 (2.5%)	1.0
ICU admission	20/69 (29%)	273/951 (29%)	.93	74/276 (27%)	.39
Recurrences	7/58(12%)	24/943(2.5%)	<.001	12/276 (4.3%)	.03
Death‡	10/69 (14%)	220/959 (23%)	.14	37/273 (16%)	.98

* Any underlying condition that would make person eligible for pneumococcal vaccination [16].

† Because of difficulty contacting homeless persons post-discharge, a much higher proportion of data is missing for homeless persons.

§ Receiving antibiotics for this episode of illness when positive blood/sterile site culture obtained.

¶ Excluding cases with hospital-acquired disease.

‡ Death during hospitalization.

** Denominators vary, because not all information is available for all cases.

†† For the age-matched analysis, each homeless case was matched to the four non-homeless cases closest in age.

Of the 69 homeless persons who presented with invasive disease, six (9%) had received pneumococcal vaccine prior to admission. Six additional patients were vaccinated during or shortly after their hospitalization. Fifteen patients (22%) were known not to have been vaccinated; data were not available (either patients did not know their history, or they could not be contacted) for the remaining 42 (61%) patients. Similarly, influenza vaccination status was available for only 20 (29%) homeless patients with invasive pneumococcal disease; 10 (50%) of these had been vaccinated during the fall prior to their infection. In contrast, a pneumococcal vaccination history was available for 74% (719 of 970) patients who were not homeless, and an influenza vaccination history for 777 (80%): 28% had received pneumococcal vaccine, and 434 (45%) had been vaccinated against influenza.

The 69 episodes of invasive disease in homeless persons occurred in 58 patients: 6 patients had two episodes of disease, and 1 patient had six episodes. The proportion of patients with recurrent disease (7 of 58, 12%) was 5 fold higher than that for housed patients during the same time period (24 of 943, 2.5%, P<.001). One of eleven recurrent episodes may have been a relapse: it occurred 54 days after the first episode, and the infecting isolates were of the same serotype. All other episodes occurred more than four months apart, and, in all cases when isolates were available for typing, were caused by isolates of different serotypes. Three additional patients with episodes of invasive disease while they were homeless had another episode of disease during a time in which they had housing. Underlying liver disease was more common in the ten patients who had recurrent episodes of disease than in other homeless patients (6 of 10, 60% versus 12 of 48 (25%), P = .055), but the two groups of patients did not differ in other characteristics. The patient with six episodes of illness was a smoker and intravenous drug user in her 50s with HIV and hepatitis C co-infection, and known hepatic cirrhosis. The case fatality rate was 7 of 58 (12%) for first episodes of disease in homeless persons, compared to 3 of 7 (43%) for second episodes (P = .06).

Of 69 episodes of invasive disease in homeless patients, 8 (12%) were not admitted to hospital, and 7 (10%) left hospital against medical advice. In comparison, 114 (12%) of housed patients were not admitted to hospital, and 2 (0.2%) left against medical advice.

### Clinical characteristics of laboratory-confirmed non-bacteremic pneumococcal pneumonia

Table 2 compares the clinical characteristics of laboratory-confirmed non-bacteremic pneumococcal pneumonia in homeless and housed adults. As for invasive pneumococcal disease, homeless persons were younger, less likely to be diabetic, more likely to have chronic liver disease and HIV infection, and much more likely to be smokers and to abuse alcohol. There were no statistically significant differences in patient characteristics or outcomes between homeless persons with invasive pneumococcal disease and those with laboratory confirmed, non-bacteremic pneumococcal pneumonia.

**Table 2:** Clinical characteristics of homeless and housed adults with laboratory-confirmed non-bacteremic pneumococcal pneumonia, Toronto, 2002–2006.

Characteristic	Homeless persons (N = 27)	All other adults (N = 317)§	P value
Median age (range)	47.4 years (26–62 yrs)	68.5 years (18–97 yrs)	<.0001
Gender (N, % male)	24/27 (89%)	208/317 (66%)	.02
Underlying illness			
Any*	24/27 (89%)	252/317 (80%)	.31
Diabetes mellitus	0	63/317 (20%)	.007
Chronic cardiac disease	5/27 (19%)	112/317 (35%)	.09
Chronic lung disease	9/27 (33%)	115/317 (36%)	.84
Cancer	4/27 (15%)	43/317 (14%)	.77
Chronic liver disease	7/27 (26%)	20/317 (6.3%)	.003
Chronic kidney disease	0	14/317 (4.4%)	.61
HIV infection	4/27 (15%)	10/317 (3.2%)	.02
Smoker	22/22 (100%)	108/283 (38%)	<.0001
Alcohol abuse	23/27 (85%)	57/316 (18%)	<.0001
Type of pneumonia			
Treated as out-patient	3/27 (11%)	36/317 (11%)	.32
Required hospitalization	21/27 (78%)	207/317 (65%)	
Nosocomial	3/17 (11%)	74/317(23%)	
Hospital length of stay† (median, range)	10 days (1–169 days)	8 days (1–106 days)	.42
Outcome/complications			
Empyema	0	4/317 (1.3%)	1.0
ICU admission	15/27 (56%)	121/317 (38%)	.12
Death‡	4/27 (15%)	43/316 (14%)	.77

* Any underlying condition that would make person eligible for pneumococcal vaccination [16].

† For patients with hospitalized with community acquired disease.

‡ Death during hospitalization.

§ Denominators vary, because not all information is available for all cases.

### Isolate characteristics

Isolates were available for 1309 of 1383 (95%) of episodes. The most frequently identified serotypes are shown in Table 3. Overall, 28 of 87 (32%) isolates associated with disease in homeless persons were of serotypes included in the 7-valent conjugate vaccine, and 72 of 87 (83%) isolates were of serotypes included in the 23-valent polysaccharide vaccine. There was no change over time in the proportion of infections in homeless persons caused by vaccines in the 7-valent conjugate vaccine (5/21, 24% of episodes in 2002/3 versus 9/26, 35% in 2006/7 were cause by these serotypes).

**Table 3:** Serotype distribution in patients with severe pneumococcal disease, Toronto, 2002–2006.

Serotype*	Overall	Invasive disease,	Invasive disease,	Non-bacteremic pneumonia,	Non-bacteremic pneumonia,
	N = 1309	Housed N = 943	Homeless N = 62	Housed N = 279	Homeless N = 25
3‡	161 (12%)	108 (11%)	2 (3.2%)	48 (17%)	3 (12%)
14†	112 (8.6%)	98 (10%)	3 (4.8%)	8 (2.9%)	3 (12%)
19F†	84 (6.4%)	52 (5.5%)	0	32 (11%)	0
4†	87 (6.7%)	73 (7.7%)	10 (16%)	2 (0.7%)	2 (8.0%)
22F‡	89 (6.8%)	63 (6.7%)	6 (9.7%)	16 (5.7%)	4 (16%)
6B†	74 (5.7%)	54 (5.7%)	0	20 (7.1%)	0
12F‡	71 (5.4%)	51 (5.4%)	16 (26%)	3 (1.1%)	1 (4.0%)
9V†	66 (5.0%)	51 (5.4%)	4 (6.5%)	10 (3.6%)	1 (4.0%)
6A	63 (4.8%)	41 (4.3%)	2 (3.2%)	20 (7.1%)	0
23F†	61 (4.7%)	44 (4.7%)	1 (1.6%)	15 (5.4%)	1 (4.0%)
7F‡	41 (3.1%)	35 (3.5%)	5 (8.1%)	3 (1.1%)	0
11A‡	37 (2.8%)	21 (2.2%)	1 (1.6%)	15 (5.4%)	0
18C†	29 (2.2%)	21 (2.2%)	1 (1.6%)	5 (1.8%)	2 (8.0%)
17F‡	12 (0.9%)	4 (0.4%)	0	6 (2.1%)	2 (8.0%)
In 7-valent conjugate vaccine	513 (39%)	393 (42%)	19 (31%)	92 (33%)	9 (36%)
In 13-valent conjugate vaccine	829 (63%)	615 (65%)	29 (46%)	172 (62%)	13 (52%)
In 23-valent polysaccharide vaccine	1042 (80%)	770 (82%)	52 (84%)	200 (72%)	20 (80%)

* Serotypes listed are those which comprise >5% of isolates from any one category of disease. During the surveillance period, there 3 episodes of invasive disease due to serotype 1 (none in homeless persons), 10 episodes due to serotype 8 (1 in a homeless person), and no episodes of disease due to serotype 5.

† Serotypes included in 7-valent conjugate and 23 valent polysaccharide vaccine.

‡ Serotypes included in 23-valent polysaccharide vaccine, but not the 7-valent conjugate vaccine.

There were no differences in rates of resistance between isolates from homeless and housed adults (data not shown). Of the 88 isolates available from episodes of illness in homeless persons: 2 (2.3%) were non-susceptible to penicillin (both with MIC = 2 µg;ml), 18 (20%) were resistant to erythromycin, 3 (3.5%) were resistant to trimethoprim-sulfamethoxazole, and 1 (1.2%) was resistant to levofloxacin.

No outbreaks of pneumonia or pneumococcal disease were identified by either shelters or the department of public health in Toronto during this five year period. However, there was some evidence in our surveillance data of clustering of episodes of illness due to particular serotypes, suggesting that transmission may have occurred within shelters or other shared accommodation For instance, all five episodes due to serotype 7F (6% of isolates from homeless were of serotype 7F, compared to 2.9% among housed cases, P = .18) occurred over an eight month period; all three episodes due to serotype 11B (3% of isolates from homeless vs 0.7% others, P = 0.04) occurred over an eight month period. The first isolate of serotype 12F (20% of isolates from homeless vs 4% others, P<.001), was identified in September 2003, and all subsequent isolates occurred in residents of the largest shelter or those living on the street. In addition, all ten isolates of serotype 22F identified in homeless persons were resistant to erythromycin (MIC> = 64 ug/ml); compared to 14 of 79 (18%) other serotype 22F strains (P<.0001).

## Discussion

Homelessness is an important and growing problem in the developed world [19]–[21]. Previous studies have documented a significant burden of illness among homeless persons due to underlying chronic medical conditions, tuberculosis, HIV infection, trauma, and mental illnesses and addictions [18], [21]–[25]. This burden of chronic illness, and crowded living conditions in shelters, would be expected to be associated with an increased incidence of invasive pneumococcal disease. Nonetheless, the high rates of pneumococcal disease identified in this and a previous smaller study in Edmonton [7], are strikingly high; only patients with AIDS, hematologic malignancies and stem cell transplants have been identified as at similar or higher risk [14], [26]. It is not possible to distinguish from this study the extent to which the increased incidence of disease is due to increased host susceptibility versus increased risk of transmission of pathogenic strains in crowded living conditions. Although most common underlying conditions do not increase the risk of invasive pneumococcal disease 30 fold [3]–[6], [27], [28], many homeless persons have more than one underlying risk factor, and there are not data on risk in persons with multiple risk factors (eg. smoking, HIV infection and liver disease).

In keeping with the hypothesis that the host susceptibility to invasive pneumococal disease of homeless persons is much higher than that of other populations, we identified a five fold increased risk of recurrent disease in this homeless population. Recurrent invasive disease is known to be associated with serious immunodeficiency, in particular multiple myeloma and other malignancies, HIV infections, and chronic liver disease [29]–[33]. Increased host susceptibility is also likely the reason why, despite the fact that homeless patients with invasive disease are much younger than housed patients, their in hospital case fatality rate is almost identical. The fact that pneumonia was more prevalent in homeless than housed persons while bacteremia without focus was less prevalent may be explained by the dramatically higher proportion of smokers in the homeless population predisposing to lung infection. [5], [9].

The very high proportion of smokers (95%) among homeless persons with invasive pneumococcal disease may also explain the fact that rates of invasive infection in homeless persons do not decrease during the summer months in parallel to decreases in invasive infection in other adults. If homeless persons have higher carriage rates of *S. pneumoniae* [10], and the high carriage rate persists in the summer, transmission might also explain the relative excess of summer disease. However, one would expect crowding to be greater in winter months when shelters are used more often.

At least four previous outbreaks of pneumococcal disease in homeless shelters have been reported [8]–[11]. Disease in these outbreaks was due to isolates of serotypes 1, 5 and/or 8, serotypes that were rare in our population. There is some evidence in our data that transmission of isolates of other serotypes is associated with disease in homeless persons. We were unable to access data regarding shelter use over time, so that it is not possible to determine whether the apparent clustering of serotypes is due to transmission within or outside of shelters. Further study to define the risks for transmission of pneumococcal disease associated with the living conditions of homeless persons is warranted.

In those patients from whom a vaccination history could be obtained, pneumococcal vaccination rates were low. The difficulty in obtaining vaccination histories, and the serotype distribution of disease make vaccination programs for homeless populations a significant challenge [34]. The coverage of 7-valent conjugate vaccines is less than 35%, and coverage with the 13-valent conjugate vaccine less than 50%. Although more than 80% of disease is caused by serotypes included in the 23-valent polysaccharide vaccine, the issue of hyporesponsiveness with repeated doses of polysaccharide vaccine in a relatively young population for whom consistent documentation of medical history is clearly of concern. [35] Further, the rate of recurrent disease suggests that this population is highly susceptible, so that vaccine efficacy may also be compromised [36], [37]. Although vaccination was temporally associated with the termination of the two pneumococcal outbreaks in homeless shelters in which it was used as a control measure [10], [11], important questions about the efficacy of vaccination in this population remain.

There are a number of limitations to this study. The estimate of incidence is based on a single census of homeless persons conducted in April 2006; there are no standardized methods for such a census, and no tested means of validating the number obtained. However, the number is consistent with previous expert estimates [15]. Further, the estimated incidence of invasive pneumococcal disease would still be of concern even if the census identified only 33–50% of the total homeless population. Census data by age for homeless persons are not available, so that it is not possible to calculate age-adjusted rates for this population. The relatively small number of cases annually means that, although there appeared to be no change in the serotype distribution of disease in homeless persons in association with the introduction of a pediatric vaccination program, our power to detect such a difference was low. The diagnostic challenge of pneumococcal pneumonia means that many more cases of non-bacteremic pneumococcal pneumonia occurred than we identified; the clinical characteristics and serotype distribution of undetected cases may be different from those in cases identified by our surveillance. Some data were not available for many of the cases occurring in homeless persons – in particular, we were not able to obtain data on prior pneumococcal vaccination in the majority of homeless persons, and we do not have information regarding stage of disease or treatment for those with HIV infection In addition, as noted before, our inability to obtain data on shelter use limited the interpretation of information regarding the potential of transmission of pneumococci in shelters.

Nonetheless, it is clear that the very high rates of invasive pneumococcal disease, the limitations of current pneumococcal vaccines, and the challenges of pneumococcal vaccination program delivery in homeless populations mean that the coordination of many different programs will be necessary to effectively reduce the burden of pneumococcal disease in this population. The provision of permanent housing and improved living conditions in crowded shelters might be expected to reduce transmission of this pathogen. Prevention and treatment programs for alcohol, smoking and substance abuse, and programs to improve HIV diagnosis and care delivery might prevent a fraction of cases; similarly, increasing influenza vaccination rates might be effective in preventing those cases secondary to influenza [38]. While all of these programs may be necessary – and all with have benefits beyond pneumococcal disease – they are also relatively expensive and difficult to implement. Thus, studies of the effect of systematic or targeted pneumococcal vaccination programs against *S. pneumoniae* in homeless populations, and the development of more effective pneumococcal vaccines for adults are both urgently needed.
